# Impact of Combined Rootstock Cultivar and Grafting Method on Growth, Yield, and Quality of Soilless-Grown Cucumber (*Cucumis sativus* L.) in a Non-Temperature-Controlled High Tunnel

**DOI:** 10.3390/plants14243792

**Published:** 2025-12-12

**Authors:** Takgoa A. Phalakatshela, Puffy Soundy, Sanele F. Kubheka, Martin M. Maboko

**Affiliations:** 1 Department of Crop Sciences, Tshwane University of Technology, Private Bag X680, Pretoria 0001, South Africa; takgoa@gmail.com (T.A.P.); soundyp@tut.ac.za (P.S.); kubhekasf@tut.ac.za (S.F.K.); 2 Department of Agriculture and Animal Health, University of South Africa, Roodepoort 1709, South Africa

**Keywords:** double-root system, early yield, non-grafted plants, plant height, scion, total yield

## Abstract

Growers rarely use the grafting method on a double-root system due to limited information on the added advantages for increased plant vigour and yield of soilless-grown cucumber (*Cucumis sativus* L.). The study aimed to investigate the effect of combining rootstock cultivar and the grafting method on the growth, yield, and quality of soilless-grown cucumber in a non-temperature-controlled (NTC) tunnel. Two rootstock cultivars, Flexifort (Flex) (*Cucurbita maxima* × *Cucurbita moschata*) and Ferro (Fer) (*C. maxima* × *C. moschata*), were grafted with scion cultivar Hoplita (H) to either single- (1R) or double- (2R) root systems, and the original scion root system was combined with either a Flexifort or Ferro rootstock (O1R) to two root systems and a non-grafted plant (Hoplita). Plants were grown in 10 L containers filled with sawdust as a growing medium. The leaf number was higher in ‘HO1RFlex’ combinations, while the non-grafted plants had a significantly lower leaf number. Thicker stem diameter was obtained from non-grafted plants. The tallest plants were obtained from HO1Fer combinations at 39, 53, and 101 days after transplanting (DAT), while non-grafted plants at 25 and 101 DAT were the shortest plants. Plants grafted to single- or double-root systems, regardless of rootstock cultivar, had higher early, marketable, and total yield compared to non-grafted cucumber. Many medium-sized fruits were obtained in ‘HO1RFlex’ combinations during the early harvest. The total soluble solids (TSSs) of cucumber juice were higher in ‘H1RFer’ while fruit mineral content was not affected by the combined rootstock cultivar and grafting method. Grafting to a double-root system using the original scion roots combined with rootstock or double rootstock had a limited effect compared to plants grafted to a single-root system. It is recommended that scion be grafted to a single-root system of either rootstock Ferro or Flexifort compared to a double-root system, particularly for the cost effectiveness of seeds and labour used in grafting, as well as for improved vegetative growth, including early marketable and total yield of cucumber. The growing containers of various sizes need further investigation to allow for the root extension and growth of grafted plants.

## 1. Introduction

Cucumber (*Cucumis sativus* L.) belongs to the Cucurbitaceae family. It is known as a high-value crop and is commonly grown worldwide under protected environments such as greenhouses [1,2]. Cucumbers are generally grown in temperature-controlled or non-temperature-controlled plastic tunnels/or greenhouses to avoid windy conditions, which cause the fruit to rub against the thorny leaves and stems of the plants, resulting in unmarketable fruit due to blemishes or scars. Cucumber fruit is a popular salad vegetable, used for fresh consumption and preservation. Nutritionally, the edible portion of the cucumber contains 0.4% protein, 2.5% carbohydrates, 0.1% fat, 7.0 mg vitamin C, 25 mg phosphorus, 10 mg calcium, and 1.5 mg iron per 100 g edible fruit [3]. It is also rich in vitamins C, E, and B groups, including folic and pantothenic acids [4,5].

In southern Africa and other countries in tropical/subtropical climates, the potential for the high productivity of tomatoes and cucumbers exists due to the high solar radiation plants receive [6,7]. However, efficient production of cucumbers in South Africa has been limited by many challenges, including biotic and abiotic stresses, especially during high temperatures and drought. Climate change, associated with extreme weather conditions such as high temperatures, has been reported in recent years to negatively affect the growth performance of vegetable crops when grown in greenhouses/NTC tunnels [6,8]. The detrimental effects of abiotic stress on cucumber physiology hamper productivity [9,10]. Cultivation in naturally ventilated greenhouses during summer often results in ambient air that exceeds the optimum temperature for many crops. The combination of excessive heat and humidity imposes abiotic stress, disrupting key plant physiological processes [7]. Fluctuations in air temperature in greenhouses cause heat stress (ambient temperature over 30 °C) and induce leaf and stem wilting, which have a significant negative effect on average plant growth, yield, quality, and photosynthesis in cucumbers [6]. Wang et al. [11] indicated that heat stress induces physiological changes and changes in gene expression, including a reduction in photosynthetic function and a reduction in water and nutrient uptake [12]. The optimal temperature requirement for the growth and development of cucumber ranges from 18 to 24 °C. A study by Kumar et al. [13] reported that grafting can be considered as an alternative to develop plants that can maintain a proper growing schedule and adapt to unfavourable environments without losing their yield potential.

Grafting is a union of two or more pieces of living plant tissue that grow as a single plant [14]. Cultivars used as scions become tolerant to stresses by utilising improved rootstocks that are resistant to abiotic stresses, such as salinity [11], water stress [15], heat stress [6], and soilborne diseases [16], consequently enhancing crop production. Grafted plants grow more vigorously with strong root systems, resulting in a higher water and nutrient uptake rate, larger leaf size, and higher net assimilation rate of CO_2_ compared with non-grafted plants [17]. Several studies have demonstrated that cucumber grafting can increase total yields [18]. The performance of grafted plants depends on rootstock–scion interactions and the cultivation method [19]. Similar results across four growing seasons demonstrated that grafted cucumbers had significantly earlier harvests compared to non-grafted cucumbers [20]. Substantial differences in the quantitative response between grafted and non-grafted cucumber plants when grown in a soilless medium have been reported [21]. Previous studies have reported an increase in vegetative growth, early flowering, and early yield in grafted plants compared to non-grafted cucumber plants [22]. However, optimal cucumber plant vegetative growth, yield, and fruit quality are based on both shoot and root genotypes [18]. Thus, the introduction of the grafting method has been widely used to enhance yield and minimise the negative effects of abiotic stresses caused by low and high temperatures, as described by Venema et al. [23]. Ahmad et al. [24] concur that climatic and environmental changes encourage researchers to explore new grafting technologies and different production systems, like the use of a soilless medium as a solution for crop production to supply food demand worldwide. Studies have been conducted to evaluate the growth, yield, and quality of grafted cucumbers in high NTC plastic tunnels, with limited information on the performance of different rootstock cultivars using different grafting methods.

Generally, cucumber growers rely on a single rootstock grafted to a scion, with little or no information on plants grafted with double rootstock or rootstock combined with the scion root system to increase root volume. This study aimed to compare different rootstocks and grafting methods to improve the growth, yield, and quality of cucumbers grown in a soilless medium under a non-temperature-controlled plastic tunnel. The results of this study could help soilless cucumber growers identify rootstock and grafting method combinations that yield high-quality produce when grown in a soilless medium within an NTC plastic tunnel. Plants with double-root systems are expected to increase root volume and outperform single-root systems in a non-temperature-controlled tunnel for improved early yield, total, and marketable yield, with improved fruit mineral content.

## 2. Materials and Methods

### 2.1. Study Area

**Figure 1:**
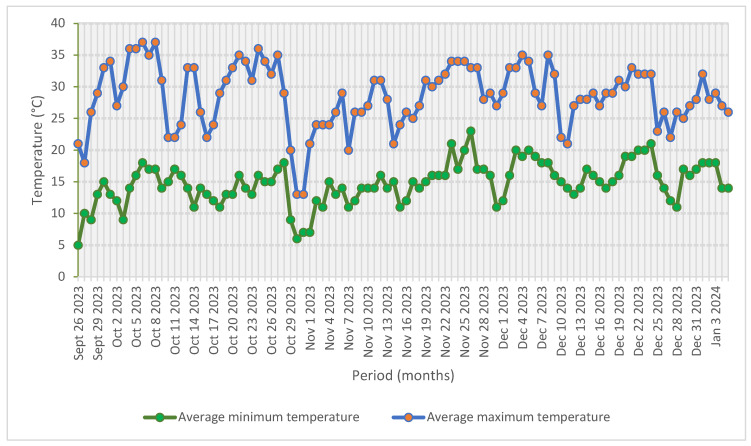
Average minimum and maximum temperature data over a four-month period during the experimental study (AccuWeather Inc., Pretoria, South Africa, 2024).

The study was conducted from September 2023 to January 2024 in a non-temperature-controlled (NTC) plastic tunnel (10 m width × 30 m length × 4.2 m height) at Latonsboer farm, Wallmansthal, north of Pretoria, South Africa (latitude 25°35′ S, longitude 28°15′ E, 1203 m above sea level). The NTC plastic tunnel relied on natural ventilation using a flap and door system that could be opened on each side of the structure to remove heat load. The NTC plastic tunnel was covered with 200 μm-thick light-diffusive UV-resistant polyethylene plastic (Greenhouse Technologies, Johannesburg, South Africa). The daily mean temperature ranged from 5 °C (minimum) to 37 °C (maximum) during the trial period from September 2023 to January 2024 (Figure 1).

### 2.2. Study Design

Two interspecific rootstock cultivars, Flexifort (*Cucurbita maxima* × *Cucurbita moschata*) and Ferro (*Cucurbita maxima* × *C. moschata*), and one scion cultivar, Hoplita, were used in this study and their characteristics are displayed in Table 1. The two rootstock cultivars were chosen because they are the most popular among commercial growers/nurseries for cucumber grafting due to their vigour and resistance to soilborne diseases, while Hoplita is a commercial summer season cultivar grown in South Africa.

**Figure 2:**
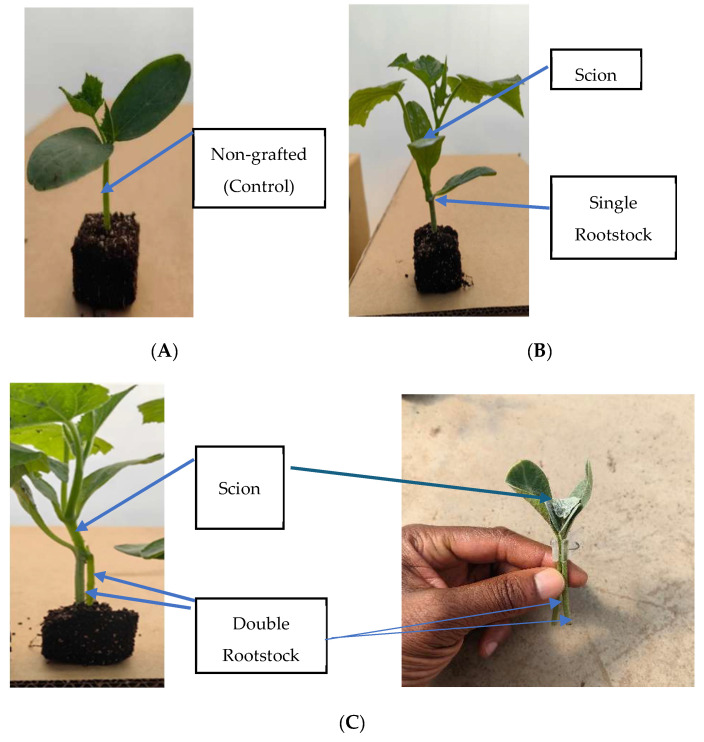
Different grafting methods in cucumber plants: (**A**) non-grafted plant; (**B**) 1R = single-grafted plant; (**C**) 2R = double-grafted plant.

Cucumber seeds were sown in white peat moss (4 × 4 cm blocks) and covered with coco-peat after seeding. Scion seeds were planted two days earlier than the rootstock seeds to allow for a good combination of seedlings. Seeds were placed in a germination room at 26.5 °C for 3 days. Thereafter, seedlings were transferred to a greenhouse, where the temperature was 27 ± 1/17 ± 1 °C (day/night) at a relative humidity of 40/60% (day/night) for 5 days. Seedlings were then grafted on day 8 after seeding. The scion and the rootstock were removed from the blocks/seedling trays by cutting them off at the soil surface to separate the roots. The rootstock and scion stock were cut slanted at 35–45° downward and upward, respectively [25,26]. For the use of two rootstocks or the original scion stock combined with a single rootstock, a cleft method was used to achieve a double-root system per plant. The two separate rootstocks were cut downward with a razor blade, creating a V-shaped cut between the cotyledons. A similar procedure was followed when using a single rootstock combined with the original scion stock. A V-shaped cut was made on the stem of the scion to fit the cut in between the double rootstock or between the single rootstock and the original scion stock/stem. The scion and rootstock were held with a grafting clip, as shown in Figure 2C, whereby the scion was between the rootstocks. The grafted plants were then planted in a white peat moss block by inserting the stock into the medium and were then placed in a recovery room for 6 days at a temperature of 22 to 24 °C and relative humidity of 56–58%. A fluorescent lamp with red–blue light was used as the light source, and exposed for a 16 h photoperiod, daily. Seedlings were moved from the recovery room into the greenhouse, separated to a spacing of 5 cm × 5 cm, and kept for 8 days before transplanting.

The experiment consisted of 7 treatments:

(1) Hoplita—control (HC);
(2) Hoplita grafted to 1 rootstock of Flexifort cv (H1RFlex);
(3) Hoplita grafted to 2 rootstocks of Flexifort cv (H2RFlex);
(4) Hoplita with its original scion stock combined with 1 rootstock of Flexifort (HO1RFlex);
(5) Hoplita grafted to 1 rootstock of Ferro RZ (H1RFer);
(6) Hoplita grafted to 2 rootstocks of Ferro RZ (H2RFer);
(7) Hoplita with its original scion root combined with 1 rootstock of Ferro RZ (HO1RFer).

The experiment was arranged in a randomised complete block design with four replicates.

### 2.3. Cultural Practices

Grafted and non-grafted cucumber seedlings were grown at a commercial nursery (Multigrow Nursery, Brits, South Africa). The grafting method used on plants grafted to one rootstock is called a splice graft, while the method used on plants grafted to double rootstock or single rootstock combined with scion root is called a cleft graft. Four hundred and seventy-six seedlings were transplanted into 10 L planting bags filled with sawdust as a growth medium. Untreated sawdust with chemicals was obtained directly from the sawmill, and pine tree wood (*Pinus sylvestris* L.) was the source of the sawdust. Plant density was maintained at 2.5 plants·m^−2^ with 17 plants per treatment and a total of 28 plots. English cucumber plants were trained to a single stem by twisting trellis twine around the main stem and fixing it to a stray wire 2.3 m away from the ground to support the plant. Side branches were pruned twice a week to maintain a single stem, as described by Maboko et al. [27]. When plants reached the horizontal wire at a height of 2.3 m, they were lowered down to allow growth to continue. The old leaves were pruned as they became yellowish. As the plants developed, removing old leaves encouraged airflow between the plants.

The pH and electrical conductivity (EC) of the nutrient solution were measured each time when the water tanks were refilled using a handheld “HANNA” EC and pH metre (HANNA Instruments, Port Louis, Mauritius). The pH was maintained at a range of 5.5 to 6.5 using nitric acid (160 mL/1000 L of water) and the EC of the nutrient solution remained at a range of 1.6 to 1.8 mS/cm. The plants were irrigated using one dripper per plant (at a discharging rate of 35 mL/min), ten times a day, every hour, each time for 2 to 10 min (equivalent to 0.7 to 3.5 L/day per plant), depending on the growth and development stages of the plants. The irrigation volume increased gradually as plants enlarged, to ensure that 10% to 15% of the applied water was leached out of the bags to reduce salt build-up in the growing medium [28].

Plants were fertigated with Hygroponic and calcium nitrate soluble fertilisers. The fertiliser’s composition and chemical concentration of Hygroponic fertiliser were as follows: N (68 mg∙kg^−1^), P (42 mg∙kg^−1^), K (208 mg∙kg^−1^), Mg (30 mg∙kg^−1^), S (64 mg∙kg^−1^), Fe (1.254 mg∙kg^−1^), Cu (0.022 mg∙kg^−1^), Zn (0.149 mg∙kg^−1^), Mn (0.299 mg∙kg^−1^), B (0.373 mg∙kg^−1^), and Mo (0.037 mg∙kg^−1^); calcium nitrate [Ca(NO_3_)_2_; and N (117 mg∙kg^−1^) and Ca (166 mg∙kg^−1^)]. Fertiliser was applied at transplanting with 600 g calcium nitrate and 600 g Hygroponic in 1000 L of water until the plants were 28 days old after transplanting. Thereafter, from 4 weeks after transplanting until the trial’s termination, plants received 800 g of calcium nitrate and 800 g of Hygroponic per 1000 L of water.

### 2.4. Plant Growth and Yield Parameters

Stem diameter, plant height, leaf chlorophyll, and leaf number were measured and recorded on 4 plants per plot, from 21 days after transplanting (DAT), thereafter every fortnight until plants reached 2.3 m height, and readings were recorded again at 101 DAT during the termination of the trial. It is worth noting that plant growth parameters coincided with the flowering, fruit set, and harvesting stages. Stem diameter was measured at 30 cm from the growing point of the plant using an electronic digital calliper (Model Proskit PD-151, Prokit’s Industries Co., Ltd., Taipei, Taiwan). Plant height was measured using a measuring tape, while the number of leaves was counted. Leaf chlorophyll content was determined using a chlorophyll metre (Model SPAD-502 plus, Konica Minolta Sensing Inc., Osaka, Japan), as described by Maboko et al. [27].

Harvesting started 31 days DAT and was completed at 101 DAT. Cucumber fruits were harvested twice every week and graded according to fruit size in terms of fruit length and fruit mass recorded using a weighing scale (Model GBK 8 bench check weighing scale, Adam equipment, Kiel, Germany). Fruit sizes were graded according to the marketable grade sizes of extra small (<25 cm), small (25 to 30 cm), medium (30 to 35 cm), large (35 to 40 cm), and extra-large (>40 cm) fruit lengths using a measuring tape. Harvested fruits were counted, while fruit mass was determined using a weighing scale. Marketable yield included fruit of small, medium, large, and extra-large sizes with uniform length and fruit diameter. Unmarketable fruit included deformed fruits with physiological disorders and fruit of an extra-small size. The first fruits that were harvested 31 to 45 DAT were regarded as early harvest, and total harvest was recorded from 31 to 101 DAT.

At 59 DAT, five fruits per plot were harvested for the measurement of fruit colour, fruit firmness, total soluble solids (TSSs), and pH of cucumber juice. At 101 DAT, four plants per treatment were then cut above the growing medium to weigh fresh and dry plant mass. Plants were oven-dried at a temperature of 72 °C for 72 h for dry mass determination.

#### 2.4.1. Fruit Colour

Fruit colour was measured using a CR-400 chromameter (Konica Minolta Sensing, Inc., Osaka, Japan), as described by Lazaro et al. [29] and Phal et al. [30]. The CR-400 chromameter was calibrated using a standard white tile. Measurements were taken on L*, a*, and b* (L* = lightness, a* = green to red, and b* = blue to yellow), as described by Bari and Khan [31].

L* = lightness (white L = 100 to black L = 0).

a* = ranging from green (−) to red (+); negative a represents green and positive a is red.

b* = ranging from blue (−) to yellow (+); negative b represent blue and positive b is yellow.

The measurement of fruit colour was recorded on the external fruit, i.e., the peel, while the inner fruit colour was taken by cutting the fruit longitudinally.

#### 2.4.2. Fruit Firmness

The firmness of cucumber fruit was measured using a fruit hardness tester (Model FHT-1122, Graigar Technology Co., Ltd., Shenzhen, China) with probe diameter of 7.9 mm and a hardness range of 0.2 to 11.0 kgf/cm^2^. Fruit firmness was measured three times along the fruit length, that is, towards the distal end, the midpoint, and towards the shoulder of the fruit. Data from each fruit sample with their respective treatments were averaged.

#### 2.4.3. Fruit Total Soluble Solids (TSSs) and pH of the Juice

Cucumber fruits were cut into pieces and placed in a laboratory blender (Model Bosch-700W Silent Mixer Black MMB43G2B, Bosch, Solingen, Germany) to produce a puree. The puree was filtered with cheese cloth to produce juice. The total soluble solid (TSS) was measured using a digital handheld refractometer (Model LH-T55, Lohand Biological, Ningbo, China). The pH of the juice was determined using a laboratory pH metre (Model SensION+pH3, Hach, Loveland, CO, USA), as described by Maboko et al. [7].

#### 2.4.4. Fruit Mineral Content

Five fruit samples per plot of uniform size were harvested and sent to Nvirotek laboratories, Hartbeespoort, South Africa, for percentage fruit moisture and fruit mineral content analysis for nitrogen (N), phosphorus (P), potassium (K), calcium (Ca), sulphur (S), magnesium (Mg), iron (Fe), zinc (Zn), copper (Cu), and manganese (Mn), on a dry weight basis. The sample analysis was carried out with an aliquot of a digested solution using inductively coupled plasma–optical emission spectrometry (ICP-OES) as described by [27].

### 2.5. Statistical Analysis

Data were subjected to analysis of variance (ANOVA) using the GenStat Windows (2022) statistical package. The significance of differences among combined treatments was tested using the least significant difference (LSD) method. Differences have been judged significant at *p* < 0.05 according to the *F*-test. The *F*-protected LSD values were calculated at a 0.05 probability level.

## 3. Results

### 3.1. Growth Parameters

**Figure 3:**
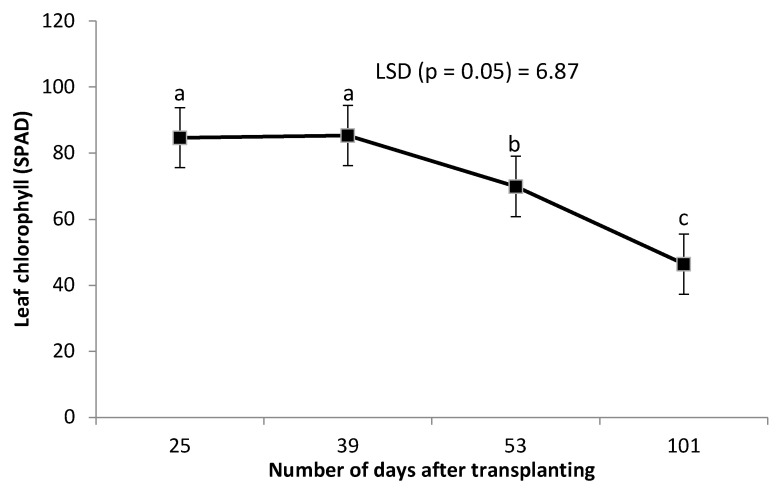
Effect of number of days after transplanting (DAT) on cucumber leaf chlorophyll content (SPAD). Points on the line with different letters are significantly different at *p* ≤ 0.05 (Fisher’s protected LSD test).

**Figure 4:**
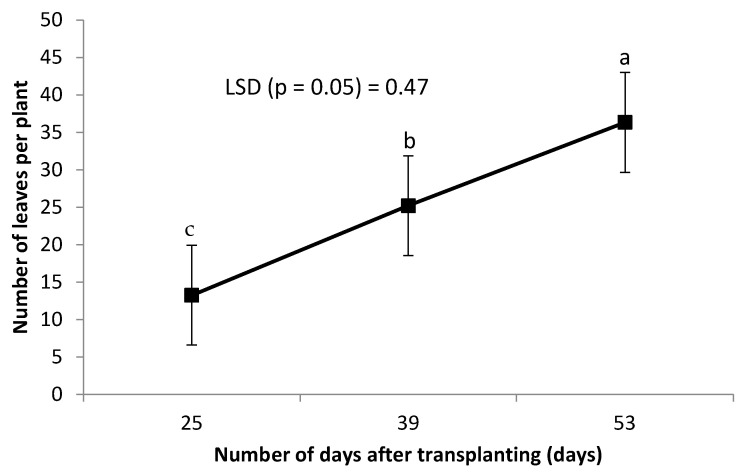
Effect of number of DAT on cucumber leaf number. Points on the line with different letters are significantly different at *p* ≤ 0.05 (Fisher’s protected LSD test).

**Figure 5:**
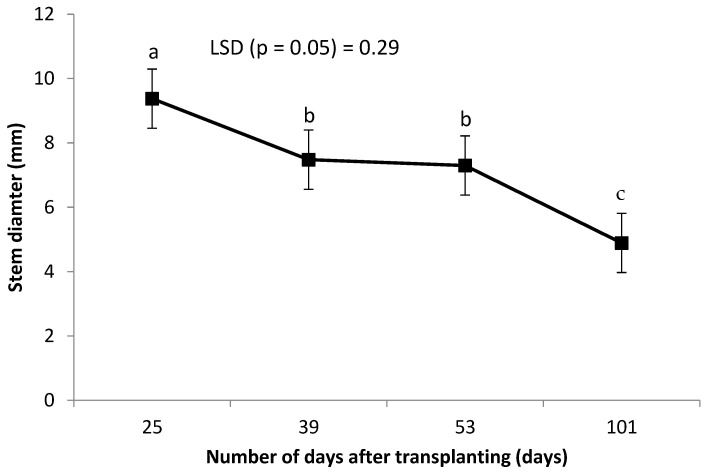
Effect of number of DAT on cucumber stem diameter (mm). Points on the line with different letters are significantly different at *p* ≤ 0.05 (Fisher’s protected LSD test).

Leaf chlorophyll was not affected by the combined grafting method and rootstock cultivar over the number of DAT (Table 2). However, leaf chlorophyll content decreased significantly with an increase in the number of DAT (Figure 3). Combined treatment of ‘HO1RFlex’ had a significantly higher leaf number, although not different from ‘HO1RFer’, ‘H2RFlex’, and ‘H2RFer’ (Table 2). Plants grafted to a single rootstock, irrespective of the rootstock cultivar, had lower leaf numbers compared to plants with double-root systems or grafted to two rootstocks (Table 2). Leaf number increased with an increase in the number of DAT (Figure 4). Generally, irrespective of the rootstock cultivar, plants grafted to two rootstocks, as well as plants grafted to a single rootstock combined with a scion root system, had higher leaf numbers (Table 2). Surprisingly, non-grafted plants had thicker stem diameters compared to other treatments, and the thinnest stems were obtained from plants grafted to single rootstocks, ‘H1RFer’ and ‘H1Rflex’ (Table 2). Stem diameter decreased with an increase in the number of DAT (Figure 5).

**Figure 6:**
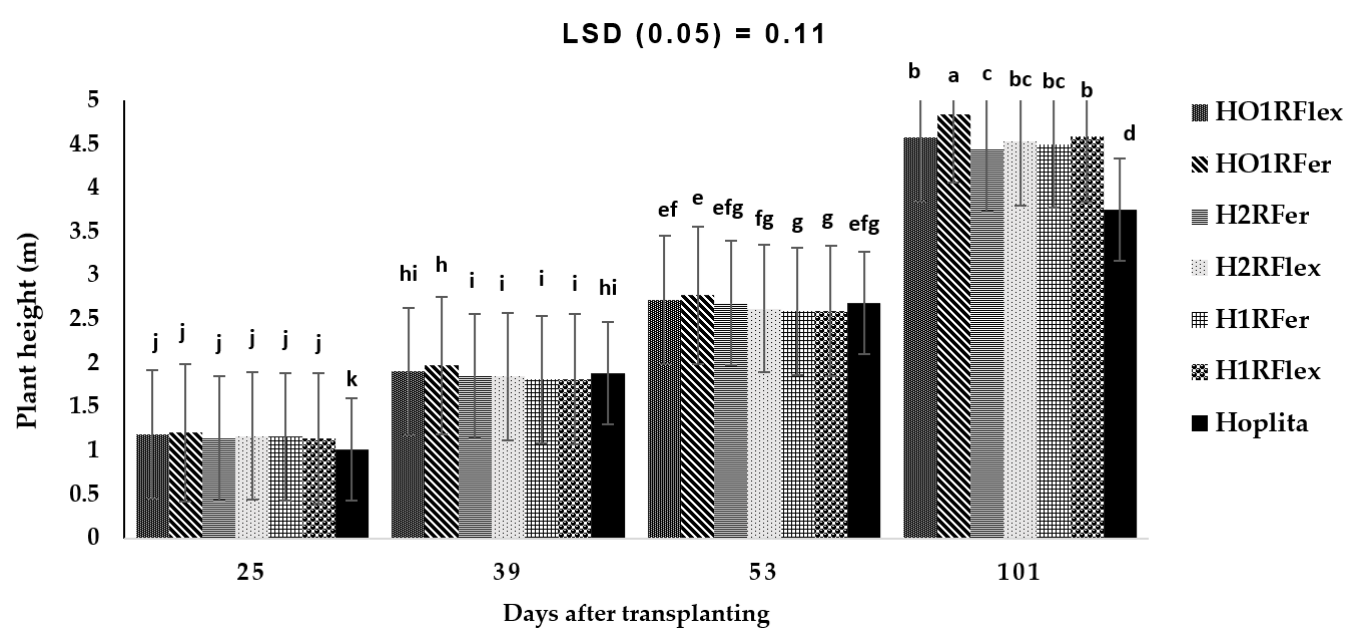
Interaction effect of combined grafting method and rootstock over the number of DAT on cucumber plant height (m). Bars with different letters are significantly different at *p* ≤ 0.05 (Fisher’s protected LSD test). Hoplita = non-grafted plant/or scion; H1RFlex = Hoplita grafted to 1 rootstock of Flexifort; H2RFlex = Hoplita grafted to 2 rootstocks of Flexifort; HO1RFlex = Hoplita (scion) original root + 1 rootstock of Flexifort; H1RFer = Hoplita grafted to 1 rootstock of Ferro; H2RFer = Hoplita grafted to 2 rootstocks of Ferro; HO1RFer = Hoplita original root system + 1 rootstock of Ferro.

On the other hand, a significant interaction was observed for the combined rootstock cultivar and grafting method, including number of days (*p* < 0.001), on cucumber plant height (Figure 6). Plant height increased with an increase in the number of days; however, at 25 DAT, irrespective of grafting method or rootstock cultivar, plants were taller than the control (Hoplita). At 39 DAT, HO1RFer and HO1Rflex treatments had taller plants compared to the other treatments. Plant height increased with an increase in the number of days, although the treatments were not significantly different from 25 to 53 DAT (Figure 6). Plants grafted to single rootstock combined with a scion root system were taller, with ‘HO1RFer’ being the tallest, while shorter plants were from ‘H2RFer’ and the shortest from ‘Hoplita’ at 101 DAT (Figure 6).

### 3.2. Yield Parameters

The first fruits harvested from 31 to 45 DAT were considered early harvests, and the total harvest was recorded from 31 to 101 DAT (Table 3). Irrespective of rootstock cultivars grafted to a single- or double-root system, grafting resulted significantly in the highest early total yield (2.3 to 2.5 kg/plant) compared to non-grafted plants (1.5 kg/plant) (Table 3). The early number of fruits harvested was higher on ‘HO1RFlex’ (5.3 fruit/plant), although not significantly different from H2RFer and ‘H1RFer’ (5 and 4.9 fruits/plant, respectively), while the lowest was found on non-grafted plants (3.1 fruit/plant) (Table 3). A similar trend was observed in early marketable yield and early marketable fruit number, with poor early marketable yield on ‘Hoplita’ (control) (Table 3). Early unmarketable yield and unmarketable fruit number were not significantly affected by the treatments (Table 3).

The highest total yield with no significant differences (6.72 to 7.14 kg/plant) was from the combined grafting method and rootstock treatments, while non-grafted plants ‘Hoplita’ (5.97 kg/plant) had the lowest total yield (Table 4). A similar trend was observed in the total fruit number, except for ‘H2RFlex’ and ‘Hoplita’, which had the lowest total fruit number (Table 4). The highest total fruit number was recorded in ‘H2RFer’ (14.90 fruits/plant), while the lowest fruit number was recorded in the control ‘Hoplita’ (12.84 fruits/plant) (Table 4). All the combined grafting and rootstock cultivar treatments performed similarly and had the highest total marketable yield with ‘H2RFer’ (6.83 kg/plant), ‘H1RFer’ (6.81 kg/plant), ‘H1RFlex’ (6.72 kg/plant), ‘HO1RFlex’ (6.47 kg/plant), ‘H2RFlex’ (6.47 kg/plant), and ‘HO1RFer’ (6.29 kg/plant), while non-grafted ‘Hoplita’ plants (5.47 kg/plant) had the lowest (Table 4). The highest total number of marketable fruits were at ‘H2RFer’ (14.2 fruit/plant), ‘H1RFer’ (13.7 fruit/plant), and ‘HO1RFlex’ (13.7 fruit/plant), followed by ‘HO1RFlex’ (13.1 fruit/plant) and ‘H1RFlex’ (13.1 fruit/plant) and the lowest was on ‘Hoplita’ (11.5 fruit/plant) (Table 4). The total unmarketable yield (0.26 to 0.46 kg/plant) and total number of unmarketable fruit (0.7 to 1.4 fruit/plant) were not affected by the combined grafting method and rootstock cultivar (Table 4).

During the early harvest, the number of extra-large-, large-, and extra-small-sized fruits, as well as deformed fruits, were not affected by the treatments (Table 5). Medium- and extra-small-sized fruits were significantly affected by the combined grafting method and rootstock cultivar (Table 5). ‘HO1RFlex’ had a high amount of early harvest medium-sized fruit, followed by other combined grafting methods and rootstock cultivar treatments, while the lowest was recorded for the control (Table 5). The number of early harvested small-sized fruits was significantly high on all grafted plants, irrespective of their grafting method and rootstock cultivar, while the control had the lowest fruit number (Table 5).

Plants grafted to ‘HO1RFlex’ had the highest total number of medium-sized fruit (8.9 fruit/plant), although it was not significantly different to ‘H2RFer’ (8.3 fruit/plant), ‘H1RFlex’ (8.1 fruit/plant), and ‘HO1RFer’ (7.8 fruit/plant), while the lowest was recorded on non-grafted plants (6.75 fruit/plant) (Table 5).

Extra-large-, large-, and extra-small-sized fruit mass, as well as deformed fruit mass of early harvest, were unaffected by the treatments (Table 6). However, the medium- and small-sized fruit mass of early harvested fruits were significantly affected by the treatments (Table 6). Early harvest of medium-sized fruit mass was high on ‘HO1RFlex’ and performed similarly to ‘H2RFer’, ‘H1RFlex’, and ‘H1RFer’, followed by ‘H2RFlex’ and ‘HO1RFer’, and the lowest was on the non-grafted plants of ‘Hoplita’ (Table 6). Early harvested yield of small-sized fruit was high on grafted plants, except for ‘H1RFlex’, while the non-grafted ‘Hoplita’ plants had the lowest yield (Table 6).

Total fruit mass according to the fruit sizes of extra-large, large, medium, small, and extra-small were not affected by treatment (Table 6). Similarly, deformed fruit mass was not significantly affected by treatment (Table 6).

The negative a* value indicates the intensity of the green colour; the positive b value describes the intensity of the yellow colour, while the L* value describes the lightness. The combined grafting method and rootstock cultivar did not affect the outer fruit skin colour for values L* and a* (Table 7). However, the b* value tended to have an intense yellowish colour on HO1RFer, HO1Rflex, and H1Rflex compared to Hoplita, including the combined grafting method and rootstock cultivar of H1Rflex, H2RFer, and H2Rflex. H2RFlex had reduced yellow intensity compared to other treatments. Inner fruit colour *L*, *a*, and *b* values were not affected by the combined grafting method and rootstock cultivar. Total soluble solids (%Brix) were significantly higher at H1RFer, followed by H2RFlex. Hoplita had the lowest TSSs, although it performed statistically similarly to other treatments, except for H1RFer and H2RFlex. The pH of the cucumber juice remained unaffected by the treatments.

Fruit mineral composition (*F* > 0.05) of the cucumber fruit, including fruit moisture content, was not affected by the combined rootstock cultivar and grafting method (Table 8). However, a tendency towards increased mineral content of Mg, K, P, Fe, Mn, Cu, and zinc was observed in cultivar Hoplita (non-grafted) compared to grafted plants, which could be explained by unrestricted root growth in a planting bag. The use of larger planting bags for grafted plants should be considered to increase the volume of growth medium, thereby increasing rooting extension and available water and nutrients for plant uptake.

## 4. Discussion

Cucumber (*Cucumis sativus* L.) is an economically important vegetable grown all over the world. In addition, consumers demand that high-quality cucumber fruit is available throughout the year, which is mainly produced under a high-tunnel system [32]. This intensive cultivation of cucumber under a high-tunnel environment can be affected by several biotic and abiotic stress factors, which may lead to a reduction in crop productivity and quality [33]. However, grafting onto appropriate rootstocks can stimulate photosynthetic processes and reduce heat stress when grown under a greenhouse production system for increased yield [33]. In this study, we investigated two rootstock cultivars, Flexifort (Flex) (*Cucurbita maxima* × *C. moschata*) and Ferro (Fer) (*C. maxima* × *C. moschata*), grafted on the scion cultivar Hoplita (HO) to either single- (1R) or double- (2R) root systems. Furthermore, the 2R systems had an original scion root system combined with a rootstock cultivar to find the best combination regarding cucumber growth, yield, and quality in comparison with the non-grafted Hoplita (scion) cultivar.

The results of plant growth, i.e., leaf number and plant height, were better for the combined grafting method and rootstock cultivar compared to Hoplita (non-grafted). The results align with those of Hernández et al. [34], who reported that vegetative growth, as measured by plant height and leaf number, was significantly higher in grafted plants compared to non-grafted plants. The high leaf number enables plants to capture more solar radiation [35], resulting in increased photosynthesis and transpiration rates, which in turn lead to higher yields [36]. Researchers [37] reported higher photosynthetic rates, improved stomatal conductance, and better water use efficiency in grafted cucumber plants, contributing to increased plant vigour. The higher leaf number on the combined rootstock and grafting method could be due to increased plant height compared to non-grafted plants [38]. Grafted plants are reported to contribute to increased nutrient uptake [39], higher root activity [40], and have a larger root system [41]. The high fruit yield could explain the reduced stem diameter on grafted plants compared to non-grafted plants, as more photosynthates could have been channelled to increase leaf number, plant height, and fruit development. In this experiment, non-grafted plants had a thicker stem diameter compared to grafted plants. Plants that were grafted to a single-root system had a tendency of reduced stem diameter compared to plants with double-root systems. The grafting technique had an effect on the stem diameter, although it did not influence yield. The improved TSSs in grafted plants could be due to a tendency for increased leaf chlorophyll and higher leaf number (Table 2), which might have contributed to increased photosynthesis and carbohydrate accumulation and metabolism [42]. Irrespective of the combined rootstock and grafting method, the plants’ roots were observed to protrude through the drainage holes of the 10 L planting bags compared to non-grafted plants (control, Hoplita). The 10 L planting bag might have restricted the root development of grafted plants, resulting in more roots coming through the drainage holes compared to the control. These observations confirm that grafted plants may improve nutrient uptake and contribute to better growth through stronger root system characteristics compared to non-grafted plants [33]. Plant roots confined to a container that restricts their growth result in competition for available oxygen and further reduce the pore space [43]. Net assimilation of summer squash was reported to have reduced the net assimilation rate under root-restricted growth [44].

Leaf chlorophyll was not affected by the combined grafting method and rootstock cultivar, though there was an increased tendency towards high leaf chlorophyll content in grafted plants. The significant decrease in leaf chlorophyll content and stem diameter with an increase in the number of days could be explained by fruit development and plant growth, as more assimilates were directed to fruit development and an increase in leaf number. Leaf chlorophyll content and N content in plants are closely related because 70% of leaf N is accumulated in chloroplasts, which produce the chlorophyll pigments [45,46]. Decreases in leaf chlorophyll concentration during the fruiting stage could be attributed to chlorophyll dilution in growing leaves or to the remobilisation of chlorophyll from leaves to fruits [47]. Leaf chlorophyll is a pigment that provides the green character of plants and occupies a significant role in the photosynthetic capacity [2,48,49] as a central exchange of carbon, water, and energy [46] for growth, to achieve high commercial cucumber yield [50].

All plants subjected to the combined rootstock cultivar and grafting method had a higher total early yield compared to non-grafted plants. Combining the scion root system with Flexifort rootstock (HO1Rflex) tended to have a higher total number of early fruits harvested, early marketable yield, and fruit number, while non-grafted plants had the least early total and marketable yield. Early harvest is advantageous for cucumber growers because it enables them to supply cucumber fruits to the market earlier and to obtain a premium price. High early yield on grafted plants could be due to the vigorous growth of the plant, thus leading to increased leaf number and plant height. Similar findings were reported by Guan et al. [20], who grafted cucumbers with squash interspecific hybrid rootstock and significantly increased vine growth and increased early season yields compared to non-grafted plants. Total and marketable yield were higher on grafted plants, irrespective of the combined rootstock cultivar and grafting method. Physiologically, grafted plants had a tendency for increased leaf chlorophyll content, which is related to effective photosynthesis, and thereby supply assimilates for fruit development, and might have contributed to the early yield and higher total yield.

Fruit size is an essential yield and quality trait for breeding and can also be a classifying feature to define different market classes of cucumbers [51]. A significant difference between grafted and non-grafted cucumber in terms of fruit length was observed, where ‘HO1RFlex’ had the greatest number of medium-sized fruit, followed by the other combined grafting method and rootstock cultivar treatments, while the lowest was recorded for non-grafted plants. Similarly, the grafting of cucumber with suitable rootstocks could be a significant factor that affects the variation in early yield [52] to obtain medium-sized fruit for the early market. However, the scion used (Hoplita) seems to produce medium-sized fruit, and further studies should be carried out on high-yielding scion cultivars that yield large to extra-large-sized fruit. The medium-sized fruit from Hoplita could be explained by its genetic makeup.

The combined grafting method and rootstock cultivar tended to have a limited effect on the b* value of the cucumbers’ external fruit colour. However, H2RFlex had reduced yellow intensity compared to the other treatments. The high b* value observed on HO1RFer, HO1Rflex, and H1Rflex compared to Hoplita, H1Rflex, H2RFer, and H2Rflex might be attributed to fruits that are harvested at an advanced stage of development, which may result in the rapid degradation of chlorophyll [53]. Generally, the low (b*) value is preferred due to the retention of a greener fruit colour. Lin et al. [54] observed that skin colour can be associated with fruit quality and appearance [55]. The L* and b* values of external fruit skin colour, as well as the L*, a*, and b* values of the internal fruit skin colour, were not significantly affected by the treatments. The combined rootstock and grafting method did not have a significant effect on cucumber pH; however, the results are within the standard cucumber pH range (5.70–5.94] [56].

Insignificant differences among treatments in fruit mineral content could be due to restricted root growth for the grafted plants, which limited oxygen availability in the rooting zone [43] and reduced energy for sufficient nutrient uptake by roots. However, in general, non-grafted plants had a tendency for high fruit mineral content compared to grafted plants.

## 5. Conclusions

Plants grafted to a single-root system and double stem, irrespective of rootstock cultivar, performed similarly in terms of total and marketable yield and improved plant height and leaf number. Grafting to a double-root system by using the original scion roots combined with rootstock or double rootstock had limited effect compared to plants grafted to a single-root system. It is recommended that the scion be grafted to a single-root system of either rootstock Ferro or Flexifort compared to a double-root system, particularly for improved vegetative growth, early, marketable, and total yield of cucumber. Furthermore, it will reduce the cost of planting extra seeds, spacing, inputs (growth medium, water, and nutrients), and labour for time spent on grafting double rootstocks to a scion. Containers of various sizes require further investigation, as the 10 L planting bag was observed to restrict root growth, to allow for the root extension and growth of plants grafted to a single- or double-root system.

## Figures and Tables

**Table 1:** Description of the cucurbit rootstock cultivars used in soilless growth medium in a plastic tunnel greenhouse.

	Rootstock Species	Scion	Characteristics	Seed Supplier
Flexiford	*Cucurbita maxima* × *Cucurbita moschata*		Resistance to *Fusarium oxysporum* f. sp. *melonis* (Fom: 0–2, 1.2) Resistance to *Fusarium oxysporum* f. sp. *niveum* (Fon, 0.1) Resistance to *Fusarium oxysporum* f. sp. *Cucumerinum*	Enza Zaden, Centurion, South Africa
Ferro RZ	*Cucurbita maxima* × *Cucurbita moschata*		Resistance to *Fusarium oxysporum* f. sp. *melonis* (Fom: 0–1, 2, 1.2) Resistance to *Verticillium wilt* (Va) Resistance to *Fusarium oxysporum* f. sp. *niveum* (For)	Rijik Zwaan, Krugersdorp, South Africa
Hoplita		Scion	CVYV = Cucumber vein yellowing (Cucumber vein yellowing virus) Ccu = Scab and gummosis (*Cladosporium cucumerinum*) CYSDV = Cucurbit yellow stunting disorder (Cucurbit yellow stunting disorder virus) Cca = Corynespora blight and target leaf spot (*Corynespora cassiicola*) Px = Powdery mildew (*Podosphaera xanthii*)	Seminis, Isando, South Africa

**Table 2:** Effect of combined rootstock and grafting method on leaf chlorophyll, number of leaves, and stem diameter of cucumber.

Grafting (GF)	Leaf Chlorophyll (SPAD)	Number of Leaves	Stem Diameter (mm)
HO1RFlex	70.3	26.062 a	7.196 cd
HO1RFer	69.2	25.646 ab	7.493 b
H2Rflex	72.9	25.188 abc	7.215 c
H2RFer	71.7	25.229 abc	7.116 cd
H1RFlex	74.5	24.688 c	6.960 de
H1RFer	73.6	24.917 bc	6.812 e
Hoplita	68.9	22.750 d	8.039 a
LSD 0.05	ns	0.8776	0.2311
Source of variation			
GF	0.803	<0.001	<0.001
DAT	<0.001	<0.001	<0.001
GF × DAT	0.915	0.135	0.102

Values in each column followed by different letters are significantly different at *p* ≤ 0.05 (Fisher’s protected LSD test). Hoplita = non-grafted plant/or scion; H1RFlex = Hoplita grafted to 1 rootstock of Flexifort; H2RFlex = Hoplita grafted to 2 rootstocks of Flexifort; HO1RFlex = Hoplita (scion) original root + 1 rootstock of Flexifort; H1RFer = Hoplita grafted to 1 rootstock of Ferro; H2RFer = Hoplita grafted to 2 rootstocks of Ferro; HO1RFer = Hoplita original root system + 1 rootstock of Ferro; GF = grafting; DAT = days after transplanting; ns = non-significant at *p* < 0.05.

**Table 3:** Effect of combined rootstock and grafting method on early total yield, fruit number, marketable yield, marketable fruit number, unmarketable yield, and unmarketable fruit number of cucumber.

Treatment	Early Total Yield (kg/plant)	Early Total Fruit Number/Plant	Early Marketable Yield (kg/plant)	Early Marketable Fruit Number/Plant	Early Unmarketable Yield (kg/plant)	Early Unmarketable Fruit Number/Plant
HO1RFlex	2.451 a	5.283 a	2.395 a	5.134 a	0.056	0.149
HO1RFer	2.269 a	4.765 b	2.140 b	4.544 b	0.129	0.221
H2RFlex	2.262 a	4.703 b	2.144 b	4.498 b	0.118	0.204
H2RFer	2.312 a	4.955 ab	2.214 ab	4.766 ab	0.098	0.190
H1RFlex	2.262 a	4.868 b	2.185 ab	4.695 ab	0.077	0.172
H1RFer	2.265 a	4.926 ab	2.226 ab	4.823 ab	0.039	0.103
Hoplita	1.484 b	3.118 c	1.466 c	3.074 c	0.018	0.044
LSD 0.05	0.1984	0.4324	0.2126	0.4624	ns	ns
*p* value	<0.001	<0.001	<0.001	<0.001	0.089	0.407

Values in each column followed by different letters are significantly different at *p* ≤ 0.05 (Fisher’s protected LSD test). Hoplita = non-grafted plant/or scion; H1RFlex = Hoplita grafted to 1 rootstock of Flexifort; H2RFlex = Hoplita grafted to 2 rootstocks of Flexifort; HO1RFlex = Hoplita (scion) original root + 1 rootstock of Flexifort; H1RFer = Hoplita grafted to 1 rootstock of Ferro; H2RFer = Hoplita grafted to 2 rootstocks of Ferro; HO1RFer = Hoplita original root system + 1 rootstock of Ferro; ns = non-significant at *p* < 0.05. For early harvests, fruit were harvested 31 to 45 DAT.

**Table 4:** Effect of combined rootstock and grafting method on total yield mass, fruit number, marketable yield, and unmarketable yield of cucumber.

Treatment	Total Yield (kg/plant)	Total Fruit Number/Plant	Marketable Yield Mass (kg/plant)	Marketable Fruit Number/Plant	Unmarketable Yield (kg/plant)	Unmarketable Fruit Number/Plant
HO1Rflex	6.726 a	14.43 ab	6.466 a	13.73 ab	0.260	0.699
HO1RFer	6.753 a	14.13 ab	6.290 a	13.07 b	0.463	1.059
H2Rflex	6.770 a	13.77 bc	6.465 a	13.08 b	0.305	0.684
H2RFer	7.109 a	14.90 a	6.826 a	14.19 a	0.283	0.714
H1Rflex	6.983 a	14.27 ab	6.716 a	13.56 ab	0.268	0.712
H1RFer	7.138 a	14.57 ab	6.813 a	13.71 ab	0.325	0.857
Hoplita	5.974 b	12.84 c	5.466 b	11.46 c	0.508	1.382
LSD 0.05	0.6015	1.085	0.6016	0.999	ns	ns
*p* value	0.013	0.021	0.002	<0.001	0.197	0.284

Values in each column followed by different letters are significantly different at *p* ≤ 0.05 (Fisher’s protected LSD test). Hoplita = non-grafted plant/or scion; H1RFlex = Hoplita grafted to 1 rootstock of Flexifort; H2RFlex = Hoplita grafted to 2 rootstocks of Flexifort; HO1RFlex = Hoplita (scion) original root + 1 rootstock of Flexifort; H1RFer = Hoplita grafted to 1 rootstock of Ferro; H2RFer = Hoplita grafted to 2 rootstocks of Ferro; HO1RFer = Hoplita original root system + 1 rootstock of Ferro; ns = non-significant at *p* < 0.05.

**Table 5:** Effect of combined rootstock cultivar and grafting method on early harvested, graded cucumber fruits.

Early Fruit Number/Plant						
Treatment	Extra-Large	Large	Medium	Small	Extra-Small	Deformed
HO1RFlex	0.00	0.237	3.982 a	0.915 a	0.0000	0.149
HO1RFer	0.00	0.279	3.368 b	0.897 a	0.0294	0.191
H2RFlex	0.00	0.260	3.396 b	0.842 a	0.0139	0.190
H2RFer	0.00	0.043	3.475 b	1.248 a	0.0588	0.131
H1RFlex	0.00	0.218	3.594 ab	0.883 a	0.0147	0.158
H1RFer	0.00	0.147	3.507 ab	1.169 a	0.0000	0.103
Hoplita	0.00	0.206	2.500 c	0.368 b	0.0000	0.044
LSD 0.05	ns	ns	0.4888	0.4284	ns	ns
*p* value	0	0.554	<0.001	0.012	0.062	0.589
	Total fruit number/plant					
H1RFer	1.50	2.31	7.73 bc	2.181	0.058	0.799
H2RFer	1.44	2.21	8.32 ab	2.223	0.059	0.655
H1RFlex	1.50	2.56	8.07 ab	1.431	0.044	0.667
H2RFlex	1.51	2.27	7.72 bc	1.592	0.058	0.626
HO1RFer	1.15	2.10	7.82 abc	2.000	0.147	0.912
HO1RFlex	0.96	2.04	8.87 a	1.857	0.060	0.640
Hoplita	0.74	1.88	6.74 c	2.103	0.044	1.338
LSD 0.05	ns	ns	1.122	ns	ns	ns
*p* value	0.335	0.696	0.033	0.217	0.784	0.307

Values in each column followed by different letters are significantly different at *p* ≤ 0.05 (Fisher’s protected LSD test). Extra-large (>40 cm), large (35 to 40 cm), medium (30 to 35 cm), small (25 to 30 cm), and extra-small (<25 cm) in fruit length at total yield harvest. Hoplita = non-grafted plant/or scion; H1RFlex = Hoplita grafted to 1 rootstock of Flexifort; H2RFlex = Hoplita grafted to 2 rootstocks of Flexifort; HO1RFlex = Hoplita (scion) original root + 1 rootstock of Flexifort; H1RFer = Hoplita grafted to 1 rootstock of Ferro; H2RFer = Hoplita grafted to 2 rootstocks of Ferro; HO1RFer = Hoplita original root system + 1 rootstock of Ferro; ns = non-significant at *p* < 0.05.

**Table 6:** Effect of combined rootstock cultivar and grafting method on cucumber fruit size at early harvest.

Early Yield (kg/plant)						
Treatment	Extra-Large	Large	Medium	Small	Extra-Small	Deformed
HO1Rflex	0.00	0.133	1.875 a	0.387 ab	0.0000	0.056
H2RFer	0.00	0.023	1.653 ab	0.538 a	0.0175	0.080
HO1RFer	0.00	0.162	1.588 b	0.390 ab	0.0210	0.108
H1RFer	0.00	0.087	1.664 ab	0.475 ab	0.0000	0.039
H1Rflex	0.00	0.126	1.699 ab	0.359 b	0.0051	0.072
H2Rflex	0.00	0.158	1.605 b	0.381 ab	0.0139	0.104
Hoplita	0.00	0.117	1.194 c	0.155 c	0.0000	0.018
LSD 0.05	ns	ns	* 0.269	0.1785	ns	ns
*p* value	0	0.497	<0.001	0.011	0.333	0.223
Mass (kg/plant)						
HO1RFlex	0.573	1.082	4.062	0.749	0.0209	0.239
HO1RFer	0.746	1.098	3.631	0.815	0.0534	0.409
H2RFlex	0.980	1.215	3.605	0.666	0.0265	0.278
H2RFer	0.918	1.111	3.887	0.911	0.0175	0.265
H1RFlex	1.017	1.351	3.764	0.583	0.0132	0.254
H1RFer	0.990	1.272	3.677	0.874	0.0073	0.318
Hoplita	0.451	1.010	3.168	0.836	0.0140	0.494
LSD 0.05	ns	ns	ns	ns	ns	ns
*p* value	0.237	0.739	0.058	0.194	0.337	0.266

Values in each column followed by different letters are significantly different at *p* ≤ 0.05 (Fisher’s protected LSD test). Extra-large (>40 cm), large (35 to 40 cm), medium (30 to 35 cm), small (25 to 30 cm), and extra-small (<25 cm) at early harvest. Early harvests, fruit harvested 31 to 45 DAT. Hoplita = non-grafted plant/or scion; H1RFlex = Hoplita grafted to 1 rootstock of Flexifort; H2RFlex = Hoplita grafted to 2 rootstocks of Flexifort; HO1RFlex = Hoplita (scion) original root + 1 rootstock of Flexifort; H1RFer = Hoplita grafted to 1 rootstock of Ferro; H2RFer = Hoplita grafted to 2 rootstocks of Ferro; HO1RFer = Hoplita original root system + 1 rootstock of Ferro; ns = non-significant at * *p* < 0.05.

**Table 7:** Effect of combined rootstock cultivar and grafting method on cucumber exterior skin and inner fruit colour, total soluble solids, and pH of juice.

Treatment	Outer Fruit Colour			Inner Fruit Colour			Total Soluble Solids (%Brix)	pH
	L*	a*	b*	L*	a*	b*		
Hoplita	27.88	−5.78	7.88 abc	41.7	−6.87	14.42	2.60 c	5.68
H1RFer	18.51	−4.57	7.54 bc	45.6	−7.50	16.27	3.18 a	5.71
H1Rflex	20.79	−5.75	8.59 a	36.7	−6.43	13.96	2.75 bc	5.97
H2RFer	24.41	−5.67	7.79 abc	40.9	−6.95	14.47	2.75 bc	5.48
H2Rflex	26.52	−4.47	6.88 c	39.8	−6.88	14.07	2.88 b	5.65
HO1RFer	23.72	−6.41	9.39 a	43.9	−6.86	13.84	2.63 c	5.57
HO1RFlex	23.91	−6.15	9.25 a	47.7	−7.31	15.80	2.70 c	5.67
LSD 0.05	ns	ns	1.65	ns	ns	ns	0.24	ns

Values in each column followed by different letters are significantly different at *p* ≤ 0.05 (Fisher’s protected LSD test). Hoplita = non-grafted plant/or scion; H1RFlex = Hoplita grafted to 1 rootstock of Flexifort; H2RFlex = Hoplita grafted to 2 rootstocks of Flexifort; HO1RFlex = Hoplita (scion) original root + 1 rootstock of Flexifort; H1RFer = Hoplita grafted to 1 rootstock of Ferro; H2RFer = Hoplita grafted to 2 rootstocks of Ferro; HO1RFer = Hoplita original root system + 1 rootstock of Ferro; ns = non-significant at *p* < 0.05.

**Table 8:** Effect of combined rootstock cultivar and grafting method on fruit mineral composition.

Treatment	N (%)	Ca (%)	Mg (%)	K (%)	Na (mg/kg)	S (%)	P (%)	Fe (mg/kg)	Mn (mg/kg)	Cu (mg/kg)	Zn (mg/kg)	Mo (mg/kg)	B (mg/kg)	Moisture (%)
HO1RFlex	2.943	0.683	0.347	4.36	819	0.477	1.030	76.0	39.00	6.67	46.3	1.223	30.33	97.80
HO1RFer	2.863	0.653	0.333	4.88	847	0.423	1.010	72.0	38.00	7.00	43.0	1.187	28.00	97.71
H2RFlex	2.700	0.640	0.330	4.15	825	0.447	1.023	81.0	40.67	6.00	50.0	1.067	28.33	97.82
H2RFer	2.940	0.537	0.317	4.10	585	0.380	0.987	70.0	36.67	6.00	44.7	1.227	28.33	97.80
H1RFlex	2.890	0.560	0.323	4.26	486	0.397	1.043	74.0	39.00	7.00	48.7	1.337	30.33	97.76
H1RFer	3.050	0.547	0.340	4.40	571	0.407	1.000	79.0	39.00	7.00	48.3	1.233	32.00	97.75
Hoplita	2.997	0.633	0.350	5.76	636	0.433	1.133	81.7	39.67	7.33	52.0	1.143	30.33	97.57
LSD 0.05	ns	ns	ns	ns	ns	ns	ns	ns	ns	ns	ns	ns	ns	ns
*p* value	0.671	0.635	0.709	0.290	0.921	0.822	0.065	0.675	0.864	0.717	0.373	0.096	0.353	0.589

ns = not significant, Fishers LSD. Hoplita = non-grafted plant/or scion; H1RFlex = Hoplita grafted to 1 rootstock of Flexifort; H2RFlex = Hoplita grafted to 2 rootstocks of Flexifort; HO1RFlex = Hoplita (scion) original root + 1 rootstock of Flexifort; H1RFer = Hoplita grafted to 1 rootstock of Ferro; H2RFer = Hoplita grafted to 2 rootstocks of Ferro; HO1RFer = Hoplita original root system + 1 rootstock of Ferro.

## Data Availability

The original contributions/data presented in this study are included in the article. Further inquiries can be directed to the corresponding author.
